# Inter-lung asymmetrical airway closure cause insufflation delay between lungs in acute hypoxemic respiratory failure

**DOI:** 10.1186/s13613-024-01379-y

**Published:** 2024-10-23

**Authors:** Hadrien Rozé, Eline Bonnardel, Eloise Gallo, Clément Boisselier, Pierre Khan, Virginie Perrier, Benjamin Repusseau, Laurent Brochard

**Affiliations:** 1 Réanimation Polyvalente, Centre Hospitalier Côte Basque, Bayonne, F-64100 France; 2 CHU de Bordeaux, Service d’Anesthésie-Réanimation Thoraco-Abdominale, Pessac, F-33600 France; 3 Université de Bordeaux, Talence, F-33400 France; 4 Keenan Research Centre, Li Ka Shing Knowledge Institute, St Michael’s Hospital, Toronto, Canada; 5 Interdepartmental Division of Critical Care Medicine, University of Toronto, Toronto, Canada

**Keywords:** Lung injury, Mechanical ventilation, Respiratory mechanics, Airway opening pressure, Electrical impedance tomography, Asynchrony, Protective ventilation

## Abstract

**Background:**

Electrical Impedance Tomography (EIT) can quantify ventilation in the two lungs and be used to measure the airway opening pressure (AOP) of each lung. Asymmetrical AOPs can cause inter-lung insufflation delay.

**Objectives:**

To assess the relation between AOP asymmetry and inter-lung insufflation delay at different PEEP levels.

**Methods:**

Patients with acute hypoxemic respiratory failure and airway closure were included. Low-flow pressure-volume curves and EIT signal were recorded during controlled ventilation and for some patients in pressure support ventilation.

**Results:**

23 patients were studied, *22 patients had ARDS*, 9 patients had asymmetrical airway closure with an AOP of 10 [6–13] cmH_2_0 in the sicker lung (AOP_sicker_) vs. 5 [3–9, ] cmH_2_0 in the healthier lung. During a low flow inflation, the inter-lung inflation delay was 0 [0-112]ms vs. 1450 [375–2400]ms in patients without or with asymmetrical AOPs, *p* < 0.0001. This delay was correlated to the difference of AOP between the 2 lungs, *Spearman* R^2^ = 0.800, *p* < 0.0001. During tidal ventilation, median delay was 0 [0–62] ms vs. 150 [50–355] ms in patients without vs. with asymmetry, *p* = 0.019. Setting PEEP at the crossing point of a decremental EIT-based PEEP trial decreased the inter-lung insufflation delay. During pressure support insufflation delay could still be measured and was reduced by increasing PEEP from 5 to 10 cmH_2_O in patient with asymmetrical lung injury.

**Conclusion:**

In asymmetrical airway closure, titrating PEEP can minimize inter-lung insufflation delay and can be monitored by EIT. Reducing the delay and reducing ventilation asymmetry is also feasible during pressure support ventilation when low flow inflation curves cannot be performed.

**Supplementary Information:**

The online version contains supplementary material available at 10.1186/s13613-024-01379-y.

## Background

In patients with acute hypoxemic respiratory failure, Computed Tomography (CT) has been used to reveal that loss of aeration was heterogeneously distributed within the lungs, occurring predominantly in the lower lobes and dependent lung regions under the effect of gravity, with the upper lobes remaining partially or entirely aerated in many patients [9, 12, 13]. The injury and the loss of aeration can also differ between the two lungs and electrical impedance tomography (EIT) has been shown to be an interesting tool to assesses imbalances of V_T_ distribution in critically ill patients [10]. When comparing the regional ventilation of different thoracic regions, the quantitative data provided by EIT mirrors the changes in air content revealed by dynamic CT [11]. EIT is well suited to detect and quantify asymmetrical injury of the two lungs in patients with unilateral or bilateral lung disease. It measures aeration percentages and between-lung differences in volume delivery. If lung injury is asymmetrical, global indices of respiratory mechanics obtained by the ventilator can be misleading as it has been shown for the global airway opening pressure (AOP) [1, 2]. An EIT-derived PV curve approach, where the volume change is assessed in each lung, can be used to assess unilateral airway opening pressure (AOP) of each lung [1, 3, 4].

EIT with regional impedance time curves can also directly visualize timing differences to open lung regions during a slow inflation maneuver or during tidal ventilation [5]. The presence of lung injury with asymmetrical AOPs can be responsible for inter-lung ventilation delay between the time when the global insufflation starts and the aeration of each lung. The level of PEEP may need to be adjusted above the highest AOP in order to keep the airways open and limit the risk of ventilator induced lung injury (VILI) induced by repeated opening and collapse of the sickest lung. Different levels of AOP should also result in time difference for the insufflation of each region according to the level of pressure reached. Personalisation of ventilator management in patients with asymmetrical ARDS therefore entails assessment of the lung-specifics risk for VILI and titration of PEEP [1, 2, 6, 7].

The goal of this study was to assess the relation between asymmetrical AOPs and inter-lung insufflation delay at different PEEP levels, with or without spontaneous breathing.

## Methods

This prospective study was approved by the appropriate French Ethical Committee through the National submission process (20/42, CPP Est IV, April 2020) and was registered (NCT04386720). Informed consent was obtained from each patient or their legal guardian before any procedure was performed.

The inclusion criteria included acute hypoxemic respiratory failure (*AHRF*, PaO_2_/FiO_2_ ≤ 300 mmHg) and intubation under assisted/controlled mechanical ventilation with continuous sedation and the observation of airway closure in at least one lung as previously described *with EIT* [1]. Patients could have uni or bilateral lung injury on chest Xray.

Exclusion criteria included lung resection or significant air leaks. All patients were ventilated to a V_T_ of 6 mL.kg^− 1^ of predicted body weight, in a volume-controlled mode and a constant flow of 60 L/min during tidal ventilation. As common practice in our institution for intubated patients with severe hypoxaemia, EIT monitoring was performed. A Pulmovista 500 was connected to an Evita Infinity V500 or V800 ventilator, Draeger, Germany. The baseline positive end-expiratory pressure (PEEP) had been previously adjusted by the attending physician before EIT without specific protocol. The measurements were performed within 48 h after intubation. The percentage ventilation of each lung was measured during tidal ventilation at the baseline PEEP. We termed the lung with the lowest percentage of tidal ventilation, and therefore the lowest compliance, the “sicker” ventilation lung. The other lung was considered as the “healthier” one with a higher tidal ventilation and a better compliance. Airway closure was *arbitrarily* considered asymmetrical if the difference between of AOP of the 2 lungs was at least 2 cmH_2_O. The AOP of the sicker lung was termed AOP_sicker_ and the AOP of the other lung AOP_healthier_.

**Fig. 1:**
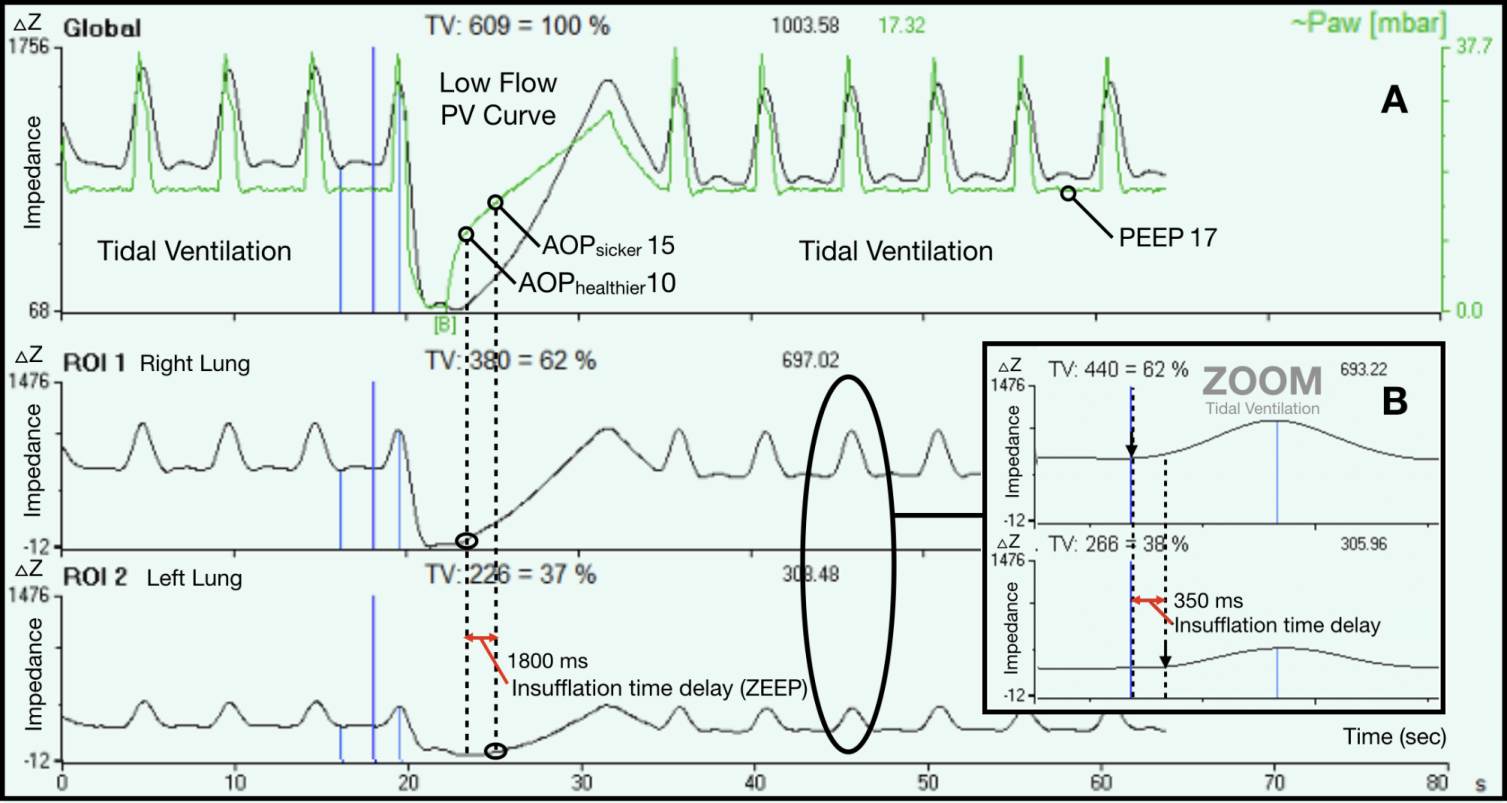
This figure illustrates a patient with asymmetrical airway closure. The impedance-time curve of each lung during tidal ventilation was recorded with Electrical Impedance Tomography monitoring. **Panel A**: The patient was under Controlled Ventilation with a PEEP of 17 cmH_2_O. The upper curve is the global Impedance-Time curve whereas the 2 following curves are the right and left lungs. The distribution of tidal ventilation is asymmetrical with 37% in the left lung. A low flow Pressure-Volume curve without PEEP was performed and shows asymmetrical airway closure between the two lungs. There was no gas entry in the right lung until a pressure of 10 cmH_2_O which represents the airway opening pressure (AOP) of the right lung. In the left lung this AOP was higher at 15 cmH_2_O. During Low Flow PV curve without PEEP the insufflation delay between the 2 lungs was 1800 ms. **Panel B**: This panel zooms in (x6) the Impedance-Time curve in order to see that during Tidal ventilation, the inflation delay was around 350 ms with a PEEP of 17 cmH_2_O

A low-flow PV curve (without PEEP) was drawn using the ventilator sensors, as previously described [8]. EIT monitoring continued during this procedure (Fig. 1 and Fig. 2 OS, online supplements).

*EIT low-flow PV curves* were performed with a flow at 6 L/min after the airway pressure signal from the ventilator was synchronised to the EIT regional changes in impedance; the impedance-time curve of each lung was recorded. All EIT-PV curves were drawn offline using dedicated software (Draeger EIT Data Analysis Tool ver. 6.3 and Excel, see online supplements, Fig. 3 OS). All PV curves were performed from zero end- expiratory pressure (ZEEP) and all signals were recorded with a low pass filter on. The curves revealed the airway closure phenomena when present and the level of AOPs of each lung as well as the insufflation delay between the two lungs during the low flow insufflation (Fig. 1OS). The AOP was the lower inflection point on each low- PV curve, as previously described [14]. AOP on EIT low flow PV curve was assess as the pressure at which some gas started to entry the lungs [15]. The curves revealed global AOP from spirometry when present and the unilateral AOPs from the EIT PV curve method in case of asymmetrical lung injury (Fig. 1).

*Impedance-Time curve with EIT* of each lung was recorded during tidal ventilation under passive volume-controlled ventilation at 60 L/min and during the low flow insufflation for AOP assessment (6 L/min). In patients with persistent asymmetrical airway closure the first day of spontaneous breathing, an EIT Impedance-Time curve of each lung was also recorded under pressure support ventilation (PSV).

Insufflation delays between the two lungs were measured on 30 tidal breaths offline with Draeger EIT Data Analysis Tool (Fig. 1). We compared inter-lung delays between patients with or without asymmetrical airway closure at ZEEP during low flow pressure volume curves, and during tidal ventilation with baseline PEEP under volume controlled or PSV. In patients under PSV, inter-lung insufflation delays on the impedance time curve were used to measure the amount of tidal volume (%V_T_) that was already insufflated in the healthier lung when the sickest lung started its delayed insufflation (see online supplement and Fig. 4).

*A decremental EIT-based PEEP trial* was performed under passive volume-controlled ventilation in order to find the best compromise of PEEP between recruitment and overdistension (PEEP_EIT_). *PEEP started at 20 cmH*_*2*_*0 and was decreased to 5 cmH*_*2*_*O by steps of 3 cmH*_*2*_*O*,* each step was 2 to 4 min until impedance time curve was stabilized. The best* level of PEEP was the crossing point of the overdistention and collapse curves during the decremental EIT-PEEP trial as previously described [16, 17].

Data were tested to see if the values *described a Gaussian* distribution using d’Agostino-Pearson omnibus normality test. Data are expressed as mean (SD) for normally distributed data, and otherwise as median [interquartile ranges, IQRs]. The t-test was used for comparisons with Gaussian distribution, Mann-Whitney for non-Gaussian distribution; we also used Pearson or Spearman correlations for normally or not distributed data. All statistical tests were two-tailed and a P-value < 0.05 was considered significant. We used Prism 6 software (GraphPad Software, La Jolla, CA, USA). Based on our previous study on asymmetrical airway opening pressure [1], and another study that showed that 1/3 of ARDS patients had airway closure [8], we targeted a sample size of 25 hypoxemic patients with airway closure in one center with medical and thoracic post operative patients in order to have at least 9 to 12 patients with significant asymmetrical airway closure.

## Results

*Twenty-five* patients with hypoxemic respiratory failure and airway closure were enrolled in the study and monitored with EIT. Their characteristics are listed in Table 1. *Only 23 patients had analysable offline EIT tracings. Twenty-two patients had ARDS.* Six patients had severe ARDS with PaO_2_/FiO_2_ < 100 mmHg and a PEEP > 5cmH_2_O and bilateral infiltrates on frontal chest X Ray [18]. Sixteen patients had moderate ARDS and two patients had mild ARDS. One patient had a unilateral pneumoniae with a PaO_2_/FiO_2_ < 200 mmHg and a PEEP > 5cmH_2_O. Some patients had thoracic surgery but without lung resection.

**Table 1:** Baseline characteristics of the patients (*n* = 23)

Characteristic	
Male gender n (%)	19 (73.0)
Age (years)	60 (13)
Body mass index (kg/m^2^)	29 (6)
SAPS II score	45 (12)
Etiology of Hypoxemic ARF: n (%)	
Post-operative Pneumonia	10 (38)
Covid-19	9 (39)
Pneumonia post Double Lung Transplantation	1 (12)
Community acquired pneumonia with pleural effusion	3 (13)

Results are expressed as number (%), or mean ± standard deviation

SAPS 2: Simplified Acute Physiology Score 2; ARF: Acute Respiratory Failure

### Symmetrical or asymmetrical AOPs

In 14 patients, airway closure was considered symmetrical with a difference in AOP between the 2 lungs on the EIT-derived PV curve less than 2 cmH_2_O. For 9 patients, airway closure was asymmetrical with a significant difference of tidal ventilation between the 2 lungs (Table 2). The mean AOPs of the sickest lungs in asymmetrical lung injury was twice the pressure for the less injured lungs. The more injured lung with less lung aeration on EIT and a lower thoraco-pulmonary compliance always had higher AOP. For 5 of the 9 patients with asymmetrical airway closure, the lowest ventilated lung was the right one. 

**Table 2:** Measurements of respiratory mechanic under passive volume-controlled ventilation in patients with symmetrical or asymmetrical airway closure

	All *N* = 23	Symmetrical *N* = 14	Asymmetrical *N* = 9	*P* value
% of ventilation sicker lung	33 [23–41]	41 [31–45]	30 [25–34]	0.021
Pplat (cmH_2_0)	27 [23–29]	27 [23–28]	27 [24–29]	0.737
PEEP (cmH_2_0)	11 [8–14]	11 [8–14]	12 [8–15]	0.820
DP (cmH_2_O)	14 [11–18]	13 [9–18]	15.0 [12.5–19.5]	0.452
V_T_ (ml.kg^− 1^ PBW)	6.0 [5.0-6.2]	6.0 [6.0-6.1]	5.8 [4.7–6.2]	0.249
Frequency (cycle/min)	22 [17–25]	24 [18–27]	23 [18–27]	0.946
Crs_spiro_ (ml.cmH_2_O^− 1^)	29 [19–39]	30 [19–39]	30 [22–52]	0.903
AOP_sicker_ (cmH_2_O)	4 [2–8]	6 [2–8]	10 [6–13]	0.021
AOP_healthier_ (cmH_2_O)	7 [2–10]	6 [2–8]	5 [3–9]	0.745
Tidal delay from ZEEP (ms)	200 [0-1050]	0 [0-112]	400 [175–1950]	< 0.0001
Tidal delay from PEEP (ms)	0 [0-170]	0 [0–0]	170 [50 355]	0.011
PaO_2_/FiO_2_ (mmHg)	130 [82–167]	115 [80–152]	145 [130–206]	0.038
pH	7.39 [7.33–7.49]	7.36 [7.29–7.44]	7.40 [7.37–7.42]	0.507
PaCO_2_ (mmHg)	45 [44–52]	47 [43–57]	44 [37–51]	0.312

Results are expressed as median [IQR]

% ventilation sicker lung: it is the % of tidal ventilation in the lung with the lowest ventilation on EIT; AOP: airway opening pressure (cmH_2_0); AOP_high replace high by healthier_ is the AOP of the healthier lung , AOP_low replace low by sicker_ is the AOP of the sicker lung ; Crs: compliance respiratory system (ml.cmH_2_O^− 1^); DP: driving pressure; V_T_: Tidal Volume (ml.kg^− 1^ PBW); Delay ZEEP: Insufflation delay between the 2 lungs on the EIT impedance-Time curve (ms) during a low flow Pressure Volume Curve at ZEEP; Delay PEEP: Insufflation delay between the 2 lungs on the EIT impedance-Time curve (ms) during tidal ventilation with baseline PEEP and a constant flow of 60 L/min

P value refers to Mann-Whitney Test : Symmetrical vs. Asymmetrical lung injury

### Inter-lung insufflation delay

In patients with symmetrical airway closure, the inter-lung inflation delay on the Impedance-Time curves of each lung during the low flow PV curve without PEEP was lower than in patients with asymmetrical lung injury (Table 2). In patients with asymmetrical airway closure, the median inter-lung insufflation delay during tidal ventilation with PEEP was higher than in patients without asymmetrical lung injury (Table 2).

During the low flow PV curve in the overall population, the delay was significantly correlated with the difference of AOP between the 2 lungs, R^2^ = 0.800, *p* < 0.0001 (Fig. 2).

The difference between the PEEP level chosen at baseline and AOP_sicker_ of the more injured lung was significantly correlated with the inter-lung insufflation delay, R^2^ = 0.572, *p* < 0.0001(Fig. 3).

### PEEPEIT in asymmetrical lung Injury

At the crossing point of overdistention and collapse curves during a decremental PEEP_EIT_ trial [17], the PEEP_EIT_ was 14 [9–16] cmH_2_O and was significantly above AOP_sicker_, (Fig. 4). At PEEP_EIT_ Pplat was 27 [24–29] cmH_2_O, DP was 15 [11–17] and overdistension was 5.7 (3.3) %.

At PEEP_baseline_ 3 patients had a PEEP below AOP_sicker_. Increasing PEEP to PEEP_EIT_ in these 3 patients, decreased the inter-lung insufflation delay with an improvement of oxygenation and limited overdistension (Table 3).

**Table 3:** Inter-lungs insufflation delays in 3 patients with airway closure and a baseline PEEP below AOP_sicker_, comparison with a PEEP from a decremental EIT-PEEP titration

Patients	1		2		3	
	PEEP_baseline_	PEEP_EIT_	PEEP_baseline_	PEEP_EIT_	PEEP_baseline_	PEEP_EIT_
PEEP (cmH_2_O)	8	17	8	12	5	10
Distribution of V_T_ (%)	10/90	22/78	55/45	53/47	18/82	23/77
AOPs_sicker/healthier (_cmH_2_O)	17/3	17/3	12/12	12/12	10/8	10/8
PF (mmHg)	140	330	212	257	190	210
Pplat (cmH_2_O)	30	27	30	30	26	29
V_T_ (ml.kg^− 1^)	7	4.4	6.5	6.5	4.5	4.5
% distension	0	6	8	11	0	5
Delay (ms)	450[400–500]	150[112–312]*	0	0	425[400–462]	300[300–350]*

*Paired t test comparison for each patient between the levels of PEEP *p* < 0.0001

### Spontaneous breathing during pressure support ventilation

In the patients with asymmetrical airway closure, three had persistent asymmetrical lung injury when switched to pressure support ventilation (PSV). With a PEEP of 5 cmH_2_O, their distribution of tidal ventilation in the sicker lung was: 10%, 20% and 30%, respectively. The difference in AOPs between each lung under passive controlled ventilation before spontaneous breathing with pressure support ventilation, were: 10, 4, and 4 cmH_2_O, respectively (Table 4).

**Table 4:** Inter-lungs insufflation delays in 3 patients under pressure support ventilation with airway closure and a baseline PEEP below the highest AOP, comparison between PEEP 5 and PEEP 10 cmH_2_O

Patients in PSV with airway closure	1		2		3	
PEEP (cmH_2_O)	5	10	5	10	5	10
Distribution of V_T_ (%)	10/90	20/80	80/20	70/30	30/70	35/65
AOPs_Right/Left_ (cmH_2_O)	10/0	10/0	3/7	3/7	10/6	10/6
PF (mmHg)	218	245	235	257	190	210
Delay (ms)	350[275–750]	200[150–250]*	450[400–500]	200[175–275]*	425[350–450]	300[300–375]*

AOPs were measured before assisted ventilation under passive controlled ventilation

*Paired t test comparison for each patient between the 2 levels of PEEP *p* < 0.0001

Inspiratory trigger delay was not present on the ventilator curves, but there was an insufflation delay between the 2 lungs on the Impedance-Time curve. With a PEEP of 10 cmH_2_O under PSV with the same level of assist, the delay decreased significantly, by 43, 56, and 29% for patients 1,2 and 3 respectively. At the same time the distribution of tidal ventilation increased in the sickest lung by 100, 50 and 17%, respectively, while expired tidal volume remained constant around 6 ml.kg^− 1^ PBW.

Under PSV, 12.9 (5.5) % of the total V_T_ was already insufflated in the healthier lung when insufflation of the sicker lung started with a delay of 408 [341–566] ms at PEEP 5 cmH_2_O. An increased of the PEEP to 10 cmH_2_O significantly reduced this % of V_T_ to 5.5 (1.6) %, *p* = 0.01.

## Discussion

Airway closure can be asymmetrical in hypoxemic respiratory failure with a higher AOP in the more injured lung. Such difference of AOPs between the 2 lungs can lead to an inter-lung insufflation delay. We show here that this delay is correlated to the difference of AOPs between the 2 lungs, the higher the difference of AOP, the higher the delay. Patients with a PEEP level above the AOP of their sickest lung will have a lower delay. Lastly, patients with persistent asymmetrical lung injury under pressure support ventilation can have an inter-lung insufflation delay, and this asynchrony can be identified by EIT and reduced by PEEP without performing a low flow insufflation curve.

Acute hypoxemic respiratory failure can result from uni or bilateral injury, and bilateral lung injury can be asymmetrical [25]. Interpretation of global lung mechanics in patients with hypoxemic respiratory failure can be misleading because it only assesses the overlapping information of several ventilatory units of different lung regions that differ in their mechanical behavior and does not necessarily reflect a mean behavior. For instance the global AOP will reflect the healthier lung, not the sickest or the mean [1]. Quasi-static PV curves have been used to derive global, respiratory mechanical indices in patients such as compliance, lower inflexion point, and end-inspiratory level of distension [14]. In unilateral lung injury, double lumen tubes have been used to measure the lower inflection point of the sick lung, but it is a difficult maneuver to perform in the ICU [6]. Regional low flow pressure volume curves with EIT in ARDS have shown that lower and upper inflection points obtained are not representative of all regions of the lungs [26]. Indeed, lower inflection points were significantly higher in the dorsal than in the ventral regions [27]. We found that in patients with asymmetrical airway closure, the lower inflection point of the low-flow ventilator PV curve reflects the AOP of the less-injured lungs. The EIT low-flow PV curve of the most severely injured lung may differ significantly with different lung compliance, V_T_, and driving pressure values [1]. This is explained by the fact that the lungs are connected in parallel. At the beginning of inflation, the less injured lung with the best compliance and the lowest airway opening pressure, will be ventilated alone until the AOP of the sickest lung is reached and this will create a delay of insufflation between the 2 lungs. We found that this inter-lung insufflation delay is well correlated to the difference of AOPs between the two lungs and also to the PEEP level chosen. The smaller the difference between PEEP and AOP of the sickest lung, the lower the inter-lung insufflation delay. We were primarily interested in the situation when lung injury between the two lungs is asymmetrical. In our previous work [1] we also measured ventral vs. dorsal but the most significant difference in AOP in this specific population was between the two lungs, right vs. left. We did not measure the regional distribution of collapse and overdistension between the two lungs at different PEEP levels but we observed that, during the decremental EIT-PEEP trial, overdistension mostly decreased in the healthier lung with the lowest AOP whereas collapse mostly increased in the sicker lung with the highest AOP.

With regard to the risk of VILI, asymmetrical AOP is of concern because the more-injured lung (with a higher AOP) will experience repeated opening and collapse (associated with a risk for atelectrauma) if the PEEP is too low [28]. In an animal model of unilateral lung injury, the major effect of PEEP was restoration of tidal ventilation to alveoli that were recruitable at higher airway pressures [29]. Notably, in another animal model of asymmetrical lung injury with transpulmonary pressure, a safe PEEP compromise (balancing recruitment with overdistension) was identified at an end-expiratory transpulmonary pressure of 0 cm H_2_O, which limited the risk of overdistention observed with higher PEEP levels [19]. In a previous report of asymmetrical ARDS associated with airway closure, we found that a PEEP above the AOP of the most injured lung improved oxygenation by reducing the ventilation/perfusion mismatch and homogenising the ventilation distribution [1]. Potentially, such a PEEP can limit the risk for repeated airway and lung tissue opening and collapse, of reabsorption atelectasis [20] and thereby potentially protects against mechanical injury and inflammation [21, 28]. PEEP can overdistend the healthy lung in unilateral pneumonia or of the less-injured lung in bilateral injury [19]. A useful compromise for protective ventilation in asymmetrical lung injury could be an increase in PEEP above the AOP of the more-injured lung and a reduction in V_T_ to maintain the plateau pressure within safe range and thus limit the risk for overdistension in the less injured lung [7]. 

At ZEEP, the greater the difference of AOPs between the lungs, the greater the inter-lung insufflation delay. A major advantage of EIT is its high temporal resolution, allowing analysis of aeration and ventilation time courses. It can focus on regional distribution and inhomogeneity of ventilation time courses (as opposed to the amount and distribution of ventilation) to quantify tidal recruitment and the influence of different PEEP levels with EIT [22]. The delay was measured from ZEEP only during the low flow PV curve which amplifies the delay, the PV curve at ZEEP revealed the asymmetrical airway closure and the levels of AOPs with significant inter-lung insufflation delays. Increasing PEEP close to the highest AOP of the sickest lung reduces this asynchrony during tidal ventilation and limit the risk of repeated opening and collapse of the more injured lung. Interestingly, we found that the crossing point of the overdistention and collapse curves during the decremental EIT-PEEP trial can be used to propose a PEEP above the AOP of the sickest lung with a limited risk of overdistension.

We found some persisting delay greater than 0 when PEEP was above the AOP of the sicker lung, we believe that the heterogeneity of lung injury in some patients might explain this with antero-posterior gradient.

*Spontaneous Ventilation.* A decremental EIT-PEEP trial is not possible under spontaneous breathing with PSV [16]. It is also not possible to do an EIT derived low flow PV curve but it is possible to use the EIT Impedance/Time curve monitoring of each lung. This curve could help to unmask asymmetrical or unilateral airway closure by showing inter-lung insufflation delay and asymmetry of ventilation, and to optimize the PEEP level. Such a PEEP can improve lung aeration of the sickest lung with a better oxygenation, but should limit the risk for repeated airway and lung tissue opening and collapse and thereby protects against mechanical injury and inflammation [21, 28]. Under PSV, a small amount of the total* V*_*T*_was already insufflated in the healthier lung when insufflation of the sicker lung started. For some patients it was close to 20% of the VT and it was a VA/Q mismatch that could explain the improvement of oxygenation with PEEP 10. Another experimental method using transpulmonary pressure and EIT has been recently proposed under PSV to find the best compromise of PEEP between collapse and overdistension and could be used in patients with asymmetrical lung injury [23].

Lastly, in spontaneously breathing patients the change in PEEP (5 vs. 10 cmH2O) could have an impact on respiratory effort. Unfortunately, we had the P0.1t for only 2 patients with the 2 levels of PEEP, it was lower with the higher PEEP (difference > 3 cmH_2_O).

Limits of the study. First, this study analyses a limited number of patients especially in patients under PSV, which limit the generalizability of the findings to a larger population. The behaviour of AOP over days in patients with acute hypoxemic respiratory failure is unknown and probably depends of the disease (uni vs. bilateral lung injury, pneumonia with pleural empyema.). We also did not measure inter-lung insufflation delays at ZEEP during tidal ventilation. The main limit of this observational study is that the outcomes of asymmetrical lung injury according to different strategies of ventilation, including PEEP titration remain unknown. So far, patients having two-quadrant involvement in chest X-ray are not enrolled into clinical trials of mechanical ventilation, although they have the same outcomes (in terms of mortality, duration of ventilation, and length of hospital stay) whether the distribution is unilateral or bilateral and therefore this category of patients deserve further research efforts [25].

## Conclusion

With EIT it is possible to obtain separate measurements of respiratory mechanics of each lung as well as distribution of ventilation, whether the injury is unilateral or bilateral but asymmetrical. Different levels of AOPs and insufflation delays can be characterized by this technique. Bedside monitoring of the EIT impedance-time curve can help to titrate PEEP in asymmetrical airway closure. The greater the difference of AOPs, the greater the inter-lung insufflation delay. The overall aim of this approach under controlled or passive ventilation is to limit the adverse effects of mechanical ventilation with poor lung aeration, and ventilator induced lung injury (repeated opening and collapse). Reducing the insufflation delay between each lung could be a simple method to adjust PEEP during pressure support ventilation when low flow inflation curves cannot be performed.

## Electronic supplementary material

Below is the link to the electronic supplementary material.

Supplementary Material 1: Additional file 1: Supplemental description of the methods: Online Supplement (OS). Additional file 2: Figure S1: Method for unilateral EIT-derived low flow PV curve assessment. Additional file 3: Figure S2: Example of low flow Pressure Volume Curves with EIT and with spirometry in a patient with asymmetrical airway closure. Additional file 4: Figure S3: Example of low flow Pressure Volume Curves with EIT and with spirometry in a patient with symmetrical airway closure. Additional file 5: Figure S4: Effect of PEEP on inter-lungs insufflation delay under pressure support, assessment of the % of VT insufflated in the healthier lung before the sicker lung was ventilated

Supplementary Material 2: This figure shows the significant correlation between the insufflation inter-lung delay in ms, measured with the EIT Impedance-Time curve of each lung during a low flow pressure volume curve without PEEP, and the difference of Airway Opening Pressure between the 2 lungs. *Spearman’s rank correlation because of the non-normal distribution*

Supplementary Material 3: This figure shows the significant correlation between the insufflation inter-lung delay in ms, measured with the EIT Impedance-Time curve during tidal ventilation with a median PEEP_baseline_ of 12 [8–15] cmH_2_O, and the difference between the PEEP level and the Airway Opening Pressure of the sickest lung (AOPsicker) in patients with or without asymmetrical lung injury. Spearman’s rank correlation because of the non-normal distribution

Supplementary Material 4: This figure illustrates individual value of PEEP_EIT_ during a decremental PEEP trial and the AOP_sicker_ of the most injured lung

## Data Availability

The data are available from the corresponding author on reasonable request.

## References

[1] Rozé H, Boisselier C, Bonnardel E, Perrier V, Repusseau B, Brochard L, et al. Electrical impedance tomography to detect Airway Closure Heterogeneity in Asymmetrical Acute Respiratory Distress Syndrome. Am J Respir Crit Care Med. 2021;203:511–5. 33030960 10.1164/rccm.202007-2937LE

[2] Bastia L, Rozé H, Brochard L. Asymmetrical lung Injury: management and outcome. Semin Respir Crit Care Med. 2022. 10.1055/s-0042-1744303 35785812

[3] Kunst PW, Böhm SH, Vazquez de Anda G, Amato MB, Lachmann B, Postmus PE, et al. Regional pressure volume curves by electrical impedance tomography in a model of acute lung injury. Crit Care Med. 2000;28:178–83. 10667519 10.1097/00003246-200001000-00029

[4] Scaramuzzo G, Spadaro S, Waldmann AD, Böhm SH, Ragazzi R, Marangoni E, et al. Heterogeneity of regional inflection points from pressure-volume curves assessed by electrical impedance tomography. Crit Care. 2019;23:119. 30992054 10.1186/s13054-019-2417-6 PMC6469223

[5] Wrigge H, Zinserling J, Muders T, Varelmann D, Günther U, von der Groeben C, et al. Electrical impedance tomography compared with thoracic computed tomography during a slow inflation maneuver in experimental models of lung injury. Crit Care Med. 2008;36:903–9. 18431279 10.1097/CCM.0B013E3181652EDD

[6] Rivara D, Bourgain JL, Rieuf P, Harf A, Lemaire F. Differential ventilation in unilateral lung disease: effects on respiratory mechanics and gas exchange. Intensive Care Med. 1979;5:189–91. 391850 10.1007/BF01683935

[7] Rouby J-J, Brochard L. Tidal recruitment and overinflation in acute respiratory distress syndrome: Yin and Yang. Am J Respir Crit Care Med. 2007;175:104–6. 17200505 10.1164/rccm.200610-1564ED

[8] Chen L, Del Sorbo L, Grieco DL, Shklar O, Junhasavasdikul D, Telias I, et al. Airway Closure in Acute Respiratory Distress Syndrome: an underestimated and misinterpreted Phenomenon. Am J Respir Crit Care Med. 2018;197:132–6. 28557528 10.1164/rccm.201702-0388LE

[9] Cressoni M, Cadringher P, Chiurazzi C, Amini M, Gallazzi E, Marino A, et al. Lung inhomogeneity in patients with acute respiratory distress syndrome. Am J Respir Crit Care Med. 2014;189:149–58. 24261322 10.1164/rccm.201308-1567OC

[10] Frerichs I, Amato MBP, van Kaam AH, Tingay DG, Zhao Z, Grychtol B, et al. Chest electrical impedance tomography examination, data analysis, terminology, clinical use and recommendations: consensus statement of the TRanslational EIT developmeNt stuDy group. Thorax. 2017;72:83–93. 27596161 10.1136/thoraxjnl-2016-208357 PMC5329047

[11] Victorino JA, Borges JB, Okamoto VN, Matos GFJ, Tucci MR, Caramez MPR, et al. Imbalances in regional lung ventilation: a validation study on electrical impedance tomography. Am J Respir Crit Care Med. 2004;169:791–800. 14693669 10.1164/rccm.200301-133OC

[12] Puybasset L, Cluzel P, Gusman P, Grenier P, Preteux F, Rouby JJ. Regional distribution of gas and tissue in acute respiratory distress syndrome. I. consequences for lung morphology. CT scan ARDS Study Group. Intensive Care Med. 2000;26:857–69. 10990099 10.1007/s001340051274

[13] Rouby J-J, Puybasset L, Nieszkowska A, Lu Q. Acute respiratory distress syndrome: lessons from computed tomography of the whole lung. Crit Care Med. 2003;31:S285–295. 12682454 10.1097/01.CCM.0000057905.74813.BC

[14] Matamis D, Lemaire F, Harf A, Brun-Buisson C, Ansquer JC, Atlan G. Total respiratory pressure-volume curves in the adult respiratory distress syndrome. Chest. 1984;86:58–66. 6734293 10.1378/chest.86.1.58

[15] Sun X-M, Chen G-Q, Zhou Y-M, Yang Y-L, Zhou J-X. Airway Closure could be confirmed by Electrical Impedance Tomography. Am J Respir Crit Care Med. 2018;197:138–41. 28719759 10.1164/rccm.201706-1155LE

[16] Costa ELV, Borges JB, Melo A, Suarez-Sipmann F, Toufen C, Bohm SH, et al. Bedside estimation of recruitable alveolar collapse and hyperdistension by electrical impedance tomography. Intensive Care Med. 2009;35:1132–7. 19255741 10.1007/s00134-009-1447-y

[17] Jonkman AH, Alcala GC, Pavlovsky B, Roca O, Spadaro S, Scaramuzzo G, et al. Lung recruitment assessed by Electrical Impedance Tomography (RECRUIT): a Multicenter Study of COVID-19 Acute Respiratory Distress Syndrome. Am J Respir Crit Care Med. 2023;208:25–38. 37097986 10.1164/rccm.202212-2300OC PMC10870845

[18] Matthay MA, Arabi Y, Arroliga AC, Bernard G, Bersten AD, Brochard LJ, et al. A New Global Definition of Acute Respiratory Distress Syndrome. Am J Respir Crit Care Med. 2024;209:37–47. 37487152 10.1164/rccm.202303-0558WS PMC10870872

[19] Bastia L, Engelberts D, Osada K, Katira BH, Damiani LF, Yoshida T, et al. Role of positive end-expiratory pressure and Regional Transpulmonary pressure in Asymmetrical Lung Injury. Am J Respir Crit Care Med. 2021;203:969–76. 33091317 10.1164/rccm.202005-1556OC

[20] Derosa S, Borges JB, Segelsjö M, Tannoia A, Pellegrini M, Larsson A, et al. Reabsorption atelectasis in a porcine model of ARDS: regional and temporal effects of airway closure, oxygen, and distending pressure. J Appl Physiol. 2013;115:1464–73. 24009007 10.1152/japplphysiol.00763.2013

[21] Tremblay L, Valenza F, Ribeiro SP, Li J, Slutsky AS. Injurious ventilatory strategies increase cytokines and c-fos m-RNA expression in an isolated rat lung model. J Clin Invest. 1997;99:944–52. 9062352 10.1172/JCI119259 PMC507902

[22] Muders T, Luepschen H, Zinserling J, Greschus S, Fimmers R, Guenther U, et al. Tidal recruitment assessed by electrical impedance tomography and computed tomography in a porcine model of lung injury*. Crit Care Med. 2012;40:903–11. 22202705 10.1097/CCM.0b013e318236f452

[23] Slobod D, Leali M, Spinelli E, Grieco DL, Spadaro S, Mauri T. Integrating electrical impedance tomography and transpulmonary pressure monitoring to personalize PEEP in hypoxemic patients undergoing pressure support ventilation. Crit Care. 2022;26:314. 36258227 10.1186/s13054-022-04198-4 PMC9578192

[24] Goligher EC, Jonkman AH, Dianti J, Vaporidi K, Beitler JR, Patel BK, et al. Clinical strategies for implementing lung and diaphragm-protective ventilation: avoiding insufficient and excessive effort. Intensive Care Med. 2020;46:2314–26. 33140181 10.1007/s00134-020-06288-9 PMC7605467

[25] Pham T, Pesenti A, Bellani G, Rubenfeld G, Fan E, Bugedo G, et al. Outcome of acute hypoxaemic respiratory failure: insights from the LUNG SAFE study. Eur Respir J. 2021;57:2003317. 33334944 10.1183/13993003.03317-2020

[26] Hinz J, Moerer O, Neumann P, Dudykevych T, Frerichs I, Hellige G, et al. Regional pulmonary pressure volume curves in mechanically ventilated patients with acute respiratory failure measured by electrical impedance tomography. Acta Anaesthesiol Scand. 2006;50:331–9. 16480467 10.1111/j.1399-6576.2006.00958.x

[27] van Genderingen HR, van Vught AJ, Jansen JRC. Estimation of regional lung volume changes by electrical impedance pressures tomography during a pressure-volume maneuver. Intensive Care Med. 2003;29:233–40. 12594585 10.1007/s00134-002-1586-x

[28] Caironi P, Cressoni M, Chiumello D, Ranieri M, Quintel M, Russo SG, et al. Lung opening and closing during ventilation of acute respiratory distress syndrome. Am J Respir Crit Care Med. 2010;181:578–86. 19910610 10.1164/rccm.200905-0787OC

[29] Blanch L, Roussos C, Brotherton S, Michel RP, Angle MR. Effect of tidal volume and PEEP in ethchlorvynol-induced asymmetric lung injury. J Appl Physiol (1985). 1992;73:108–16. 10.1152/jappl.1992.73.1.108 1506357

