# Supplementary material for: Inter-lung asymmetrical airway closure cause insufflation delay between lungs in acute hypoxemic respiratory failure
Source: Ann Intensive Care. 2024 Oct 23;14:162. doi: 10.1186/s13613-024-01379-y (PMC11499510; doi:10.1186/s13613-024-01379-y)
Supplement: Supplementary file 1 — Supplementary Material 1: Additional file 1: Supplemental description of the methods: Online Supplement (OS). Additional file 2: Figure S1: Method for unilateral EIT-derived low flow PV curve assessment. Additional file 3: Figure S2: Example of low flow Pressure Volume Curves with EIT and with spirometry in a patient with asymmetrical airway closure. Additional file 4: Figure S3: Example of low flow Pressure Volume Curves with EIT and with spirometry in a patient with symmetrical airway closure. Additional file 5: Figure S4: Effect of PEEP on inter-lungs insufflation delay under pressure support, assessment of the % of VT insufflated in the healthier lung before the sicker lung was ventilated [file 13613_2024_1379_MOESM1_ESM.docx]

**Inter-lung Asymmetrical airway closure cause insufflation delay between lungs in Acute Hypoxemic Respiratory Failure.**

**Supplement materials**

**Methods for the EIT low flow PV curve:**

Electrodes were on a belt placed on the chest between 4 and 5 intercostal spaces.

Recording was on once all 16 electrodes were checked.

The ventilator was synchronised to the EIT device in order to plot regional changes in impedance to airway pressures; all curves were drawn offline using dedicated software (Draeger EIT Data Analysis Tool ver. 6.3).

A low pass filter at 50/min was used in order to remove heart beats artefact from the EIT low flow PV curve.

Regions of interest were selected with only 2 regions, the chest was divided into two parts with (left and right) in order to have global assessment with EIT and spirometry but also two separate left and right lung assessments.

The EIT file recorded was opened with Draeger EIT Data Analysis Tool, and transformed from .eit to .asc in order to be opened with Excel and create unilateral EIT-derived Low Flow PV Curves as shown in Figure 1 OS.

EIT monitoring was recorded during the low flow Pressure Volume Curve as illustrated by the Figures 2OS and 3 OS with asymmetrical and symmetrical airway closure, respectively. During low flow inflation at 6 litres per minute, impedance variation was measured 20 times per second. Duration of inflation was dependent on pre-set tidal volume and maximal airway pressure.

The equations used with EIT for global and unilateral respiratory mechanic assessment are as follow:

***Compliances:***

- Crs_spiro global_ = V_T_/(Pplat- ZEEP or AOP_spiro_) mL.cm H_2_O^-1^

EIT was measured at this time and we calculated the respiratory system compliance of each lung (Crs_EIT one lung_):

- First, during global tidal ventilation, we calculated the ratio (ΔV/ΔZ) between V_T_ and the corresponding global ΔZ_VT_ revealed by EIT.
- Crs_EIT global_ = ΔV/ΔZ × ΔZ_global_ /(Pplat–ZEEP or AOP_global_) mL.cm H_2_O^-1^
- Crs_EIT each lung_ = ΔV/ΔZ × ΔZ _of each lung_ /(Pplat–ZEEP or AOP_of each lung_) mL.cm H_2_O^-1^

We compared the global PV curve compliance with that of spirometry and the EIT global curve.

***PEEP_EIT_ Titration***

Once the PV curves were available, we determined the extents of lung collapse and overdistension according to the variation in EIT pixel compliance during the PEEP-decremental ventilation procedure without a recruitment manoeuvre. The PEEP_EIT_ was a compromise (crossing point) between the start of alveolar collapse and commencement of overdistension (1).

**Inter-lungs insufflation delay during pressure support ventilation**

This delay was also used to assess the amount of tidal volume (in %) that was insufflated in the healthier lung when the sicker lung was ventilated with a delay under PSV. As shown on Figure 4 OS, the inter-lungs insufflation delay and the impedance time curve can be used to measure the corresponding % of tidal volume that was inflated in the healthier lung, the variation of impedance during that delay is correlated to the variation of tidal insufflation.

- First, during global tidal ventilation, we calculated the ratio (ΔV/ΔZ) between V_T_ and the corresponding global ΔZ_VT_ revealed by EIT.
- % V_T_ = 100 x (ΔV/ΔZ × ΔZ_delay._ / V_T_)

**Figure 1 OS:** Method for unilateral EIT-derived low flow PV curve assessment.


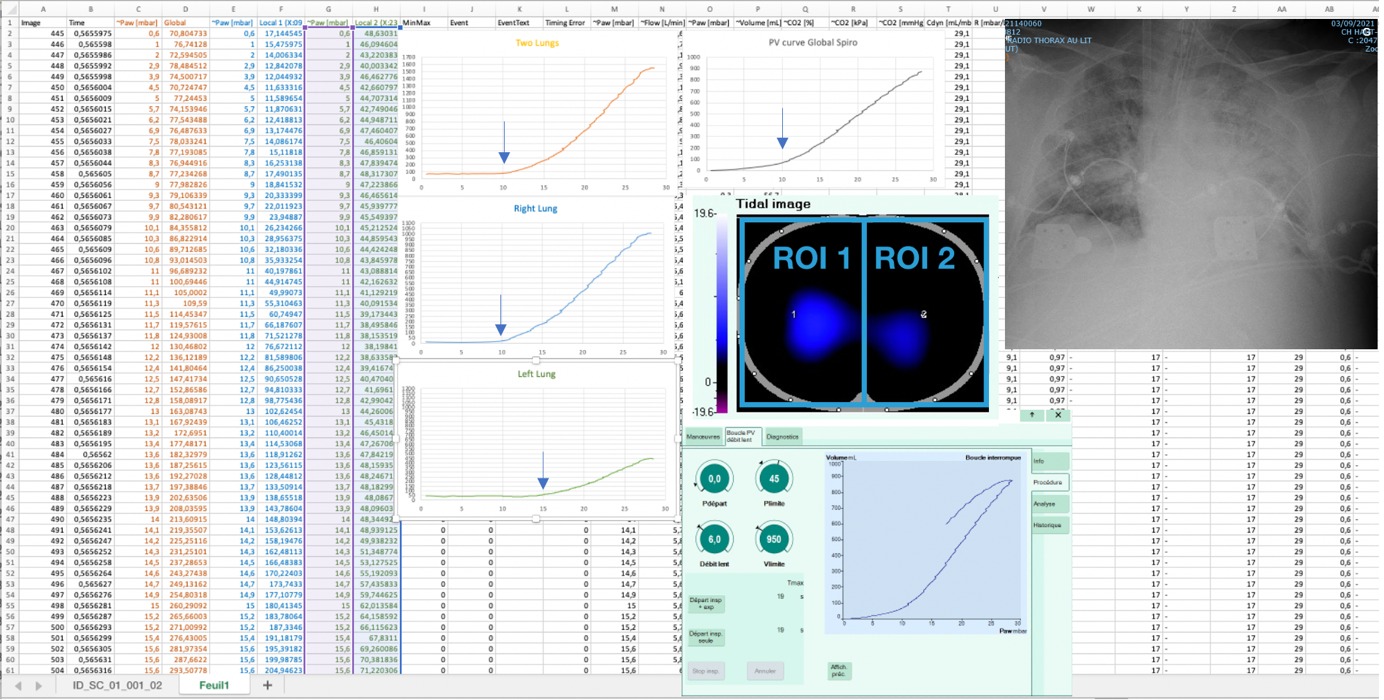


*This figure illustrates the data collected offline with EIT. The Chest X Ray illustrates the bilateral lung injuries with some asymmetrical distribution, and the position of the belt of electrodes. The classical spirometric low flow PV curves with 6 L/min of flow and no PEEP, shows a lower inflection point corresponding to an AOP of 10 cmH_2_O. EIT screen in blue illustrates the asymmetrical distribution of tidal volume during the low flow insufflation, less blue in the left lung. The excel file illustrates how we reconstructed the airway pressure and impedance variation during low flow inflation in order to create Low Flow EIT derived PV Curve in 2 different Regions of Interest: Local 1 (blue) and Local 2 (green), corresponding to the Right and Left Lungs. The global spirometric PV curve (black), the global EIT PV curve (orange) , the right EIT PV curve (blue) and the left EIT PV curve (green).*

**Figure 2 OS:** Example of low flow Pressure Volume Curves with EIT and with spirometry in a patient with asymmetrical airway closure


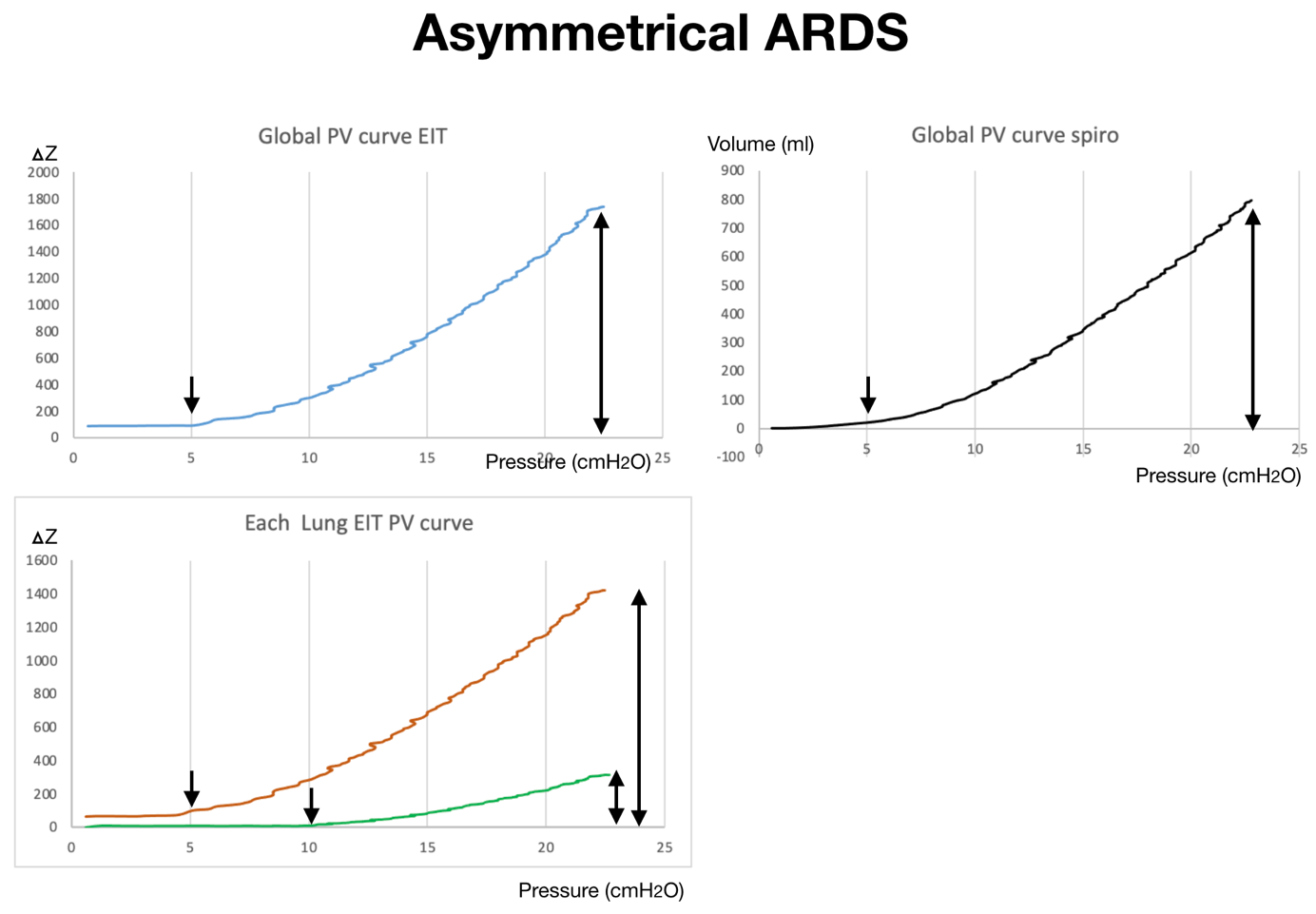


*The black global PV cure was done with the spirometer from the ventilator, showing the respiratory mechanic of the two lungs simultaneously without positive end expiratory pressure. The arrow shows a lower inflection point corresponding to the airway opening pressure (AOP) because of airway closure (here 5 cm H_2_O).*

*The colour curves were done using an EIT derived PV curve method with the variation of impedance (*$\Delta$*Z) according to the airway pressure. The blue curve is the global PV using* $\Delta$*Z from the two lungs, the shape is similar to the Global PV curve using spirometry with the same AOP. Before AOP, the blue curve is flat, there is no gas entry into the lungs, whereas on the black curve the slope corresponds to the compliance of the circuit with gas compression. The red PV curve with EIT is the less injured lung with more tidal ventilation (double arrow) and a better compliance, the AOP is the same as in the global PV curve. The green curve is the more injured lung with less tidal ventilation, a lower compliance and a higher AOP (10 cmH_2_O).*

**Figure 3 OS:** Example of low flow Pressure Volume Curves with EIT and with spirometry in a patient with symmetrical airway closure.


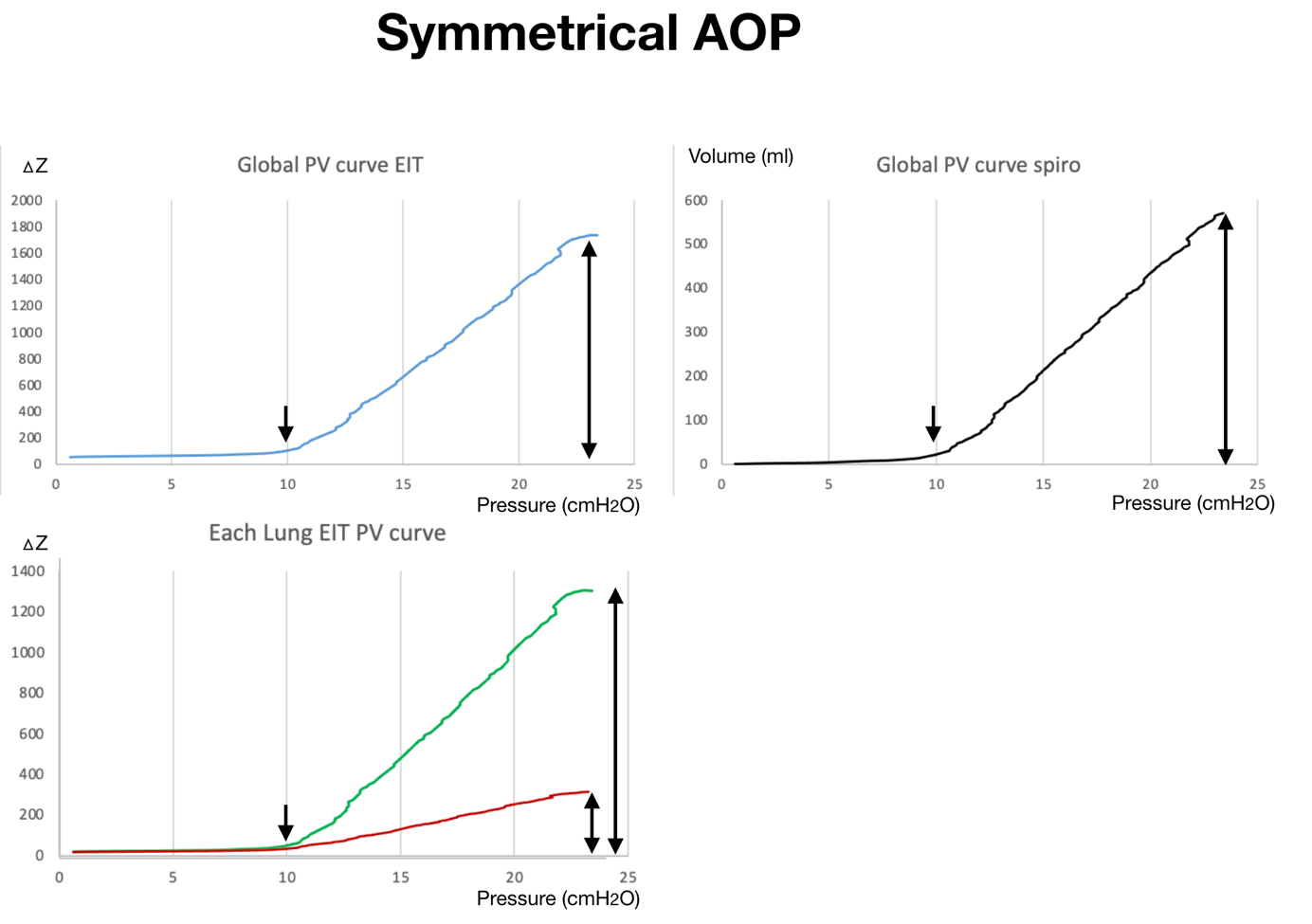


*The black global PV cure was done with the spirometer from the ventilator, showing the respiratory mechanic of the two lungs simultaneously without positive end expiratory pressure. The arrow shows a lower inflection point corresponding to the airway opening pressure (AOP) because of airway closure (here 2 cm H_2_O so not considered as significant).*

*The colour curves were done using an EIT derived PV curve method with the variation of impedance (*$\Delta$*Z) according to the airway pressure. The blue curve is the global EIT-derived PV curve using* $\Delta$*Z from the two lungs, the shape is similar to the Global PV curve using spirometry with the same slope and AOP. Before AOP, the blue curve is flat, there is no gas entry into the lungs. The tidal ventilation of the red PV curve with EIT (double arrow) is not significantly different from the green curve with a difference of less than 20 % so we considered that this ARDS is symmetrical. The slopes (compliances) of the red and green curves are not significantly difference, with the same AOP.*

**Figure 4 OS:** Effect of PEEP on inter-lungs insufflation delay under pressure support, assessment of the % of V_T_ insufflated in the healthier lung before the sicker lung was ventilated.

**
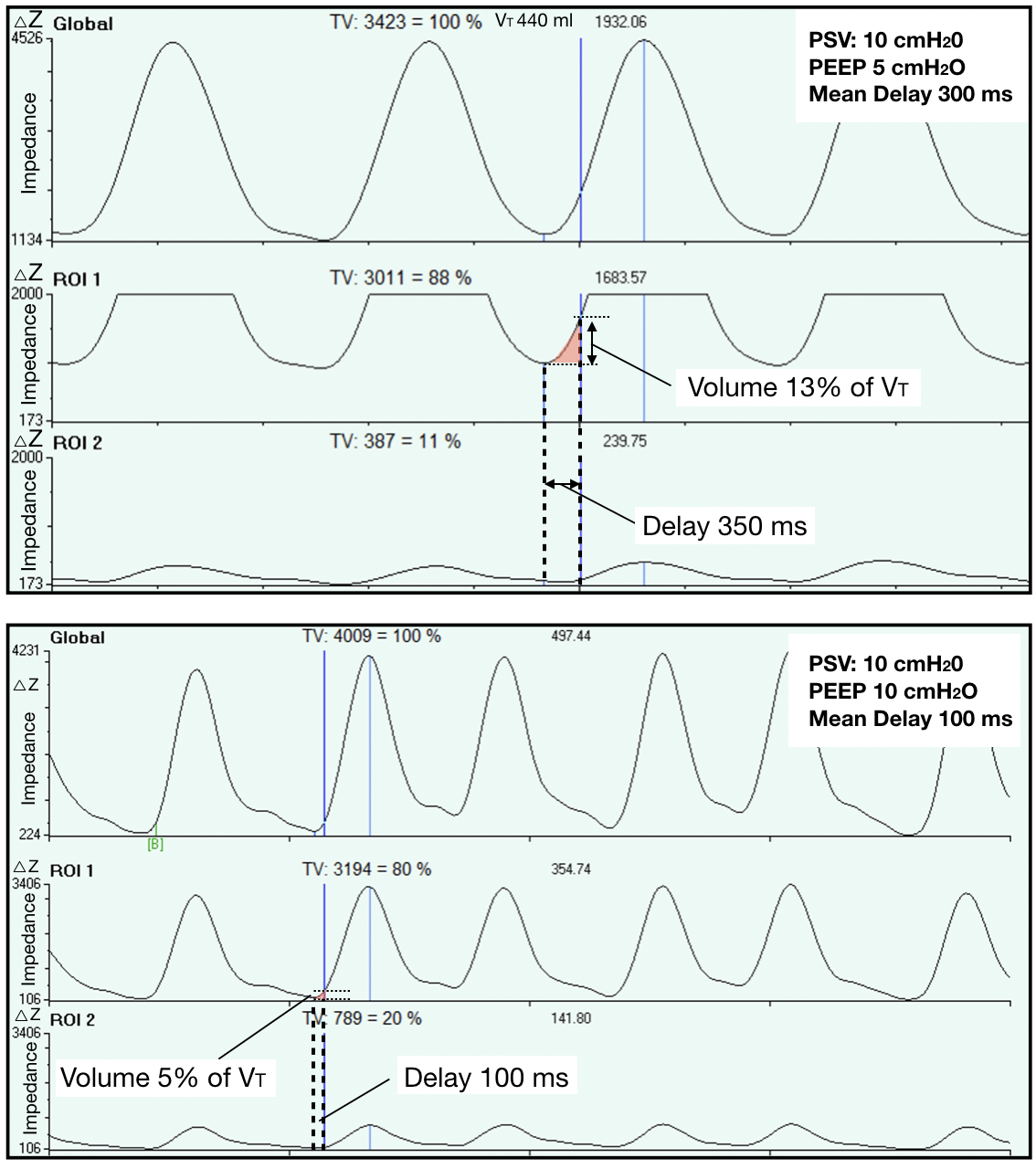
**

*This figure illustrates the inter-lungs insufflation delay due to asymmetrical airway opening pressures between the 2 lungs (ROI 1 is the right lung and ROI 2 is the left lung). An increase of PEEP from 5 to 10 significantly reduced this delay (frome 350 to 100 ms) and the % of tidal volume that was already insufflated in the healthier lung when the sicker lung was insufflated (from 13% to 5 %).*

**References:**

1. Costa ELV, Borges JB, Melo A, et al.: Bedside estimation of recruitable alveolar collapse and hyperdistension by electrical impedance tomography. *Intensive Care Med* 2009; 35:1132–1137
