# Supplementary figures and images for: Inter-lung asymmetrical airway closure cause insufflation delay between lungs in acute hypoxemic respiratory failure
Source: Ann Intensive Care. 2024 Oct 23;14:162. doi: 10.1186/s13613-024-01379-y (PMC11499510; doi:10.1186/s13613-024-01379-y)

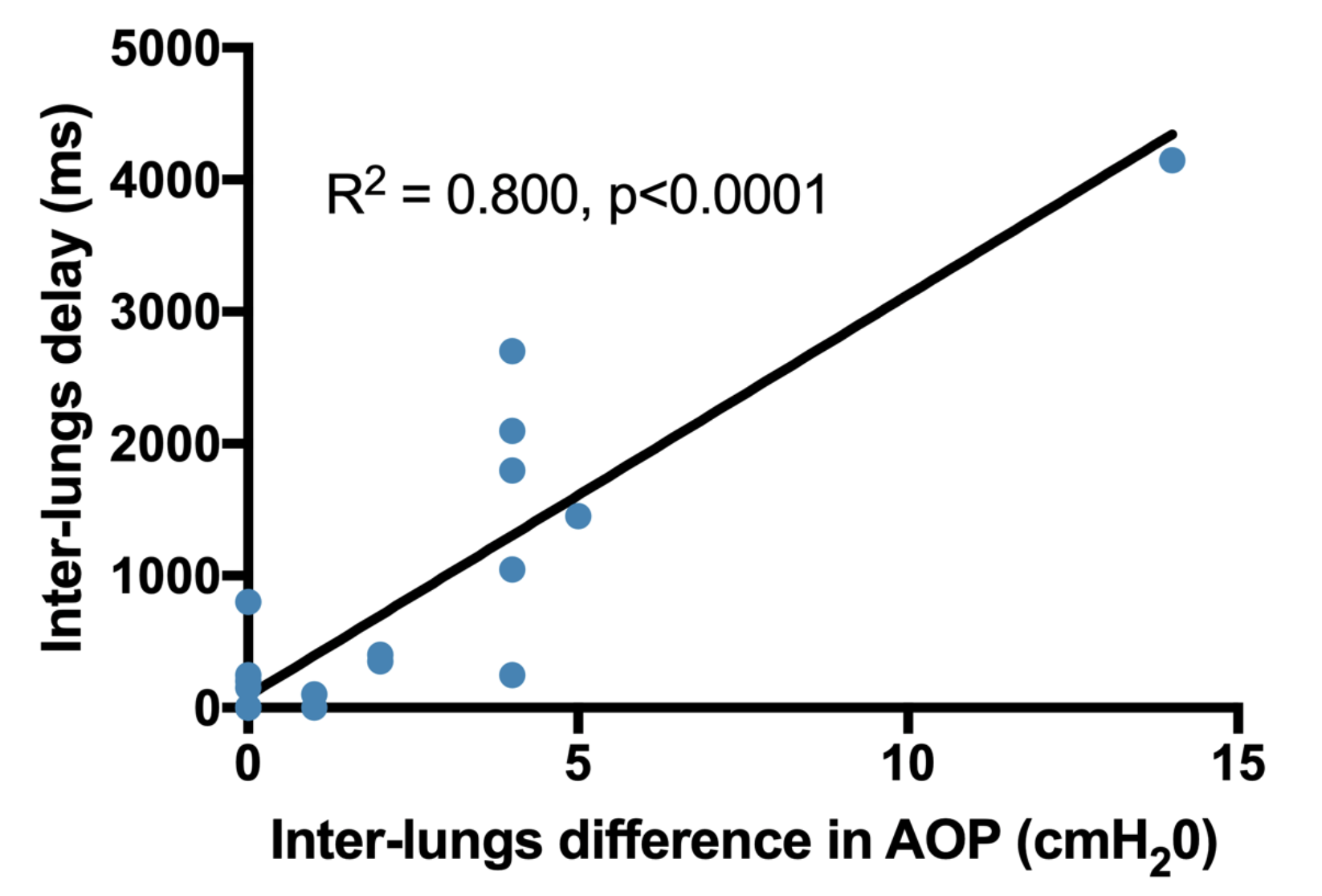

Supplement: Supplementary file 2 — Supplementary Material 2: This figure shows the significant correlation between the insufflation inter-lung delay in ms, measured with the EIT Impedance-Time curve of each lung during a low flow pressure volume curve without PEEP, and the difference of Airway Opening Pressure between the 2 lungs. Spearman’s rank correlation because of the non-normal distribution [file 13613_2024_1379_MOESM2_ESM.png]

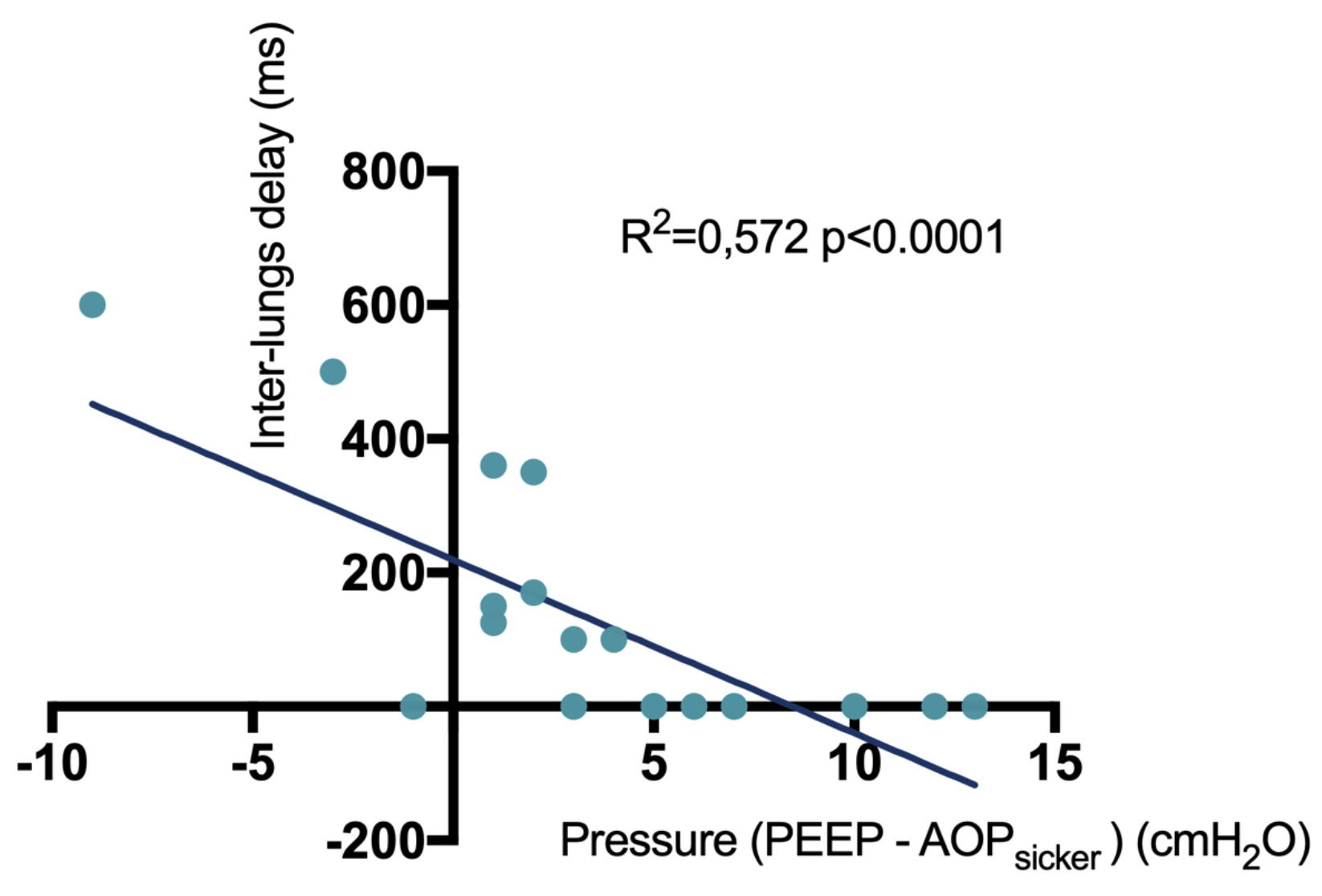

Supplement: Supplementary file 3 — Supplementary Material 3: This figure shows the significant correlation between the insufflation inter-lung delay in ms, measured with the EIT Impedance-Time curve during tidal ventilation with a median PEEPbaseline of 12 [8–15] cmH2O, and the difference between the PEEP level and the Airway Opening Pressure of the sickest lung (AOPsicker) in patients with or without asymmetrical lung injury. Spearman’s rank correlation because of the non-normal distribution [file 13613_2024_1379_MOESM3_ESM.png]

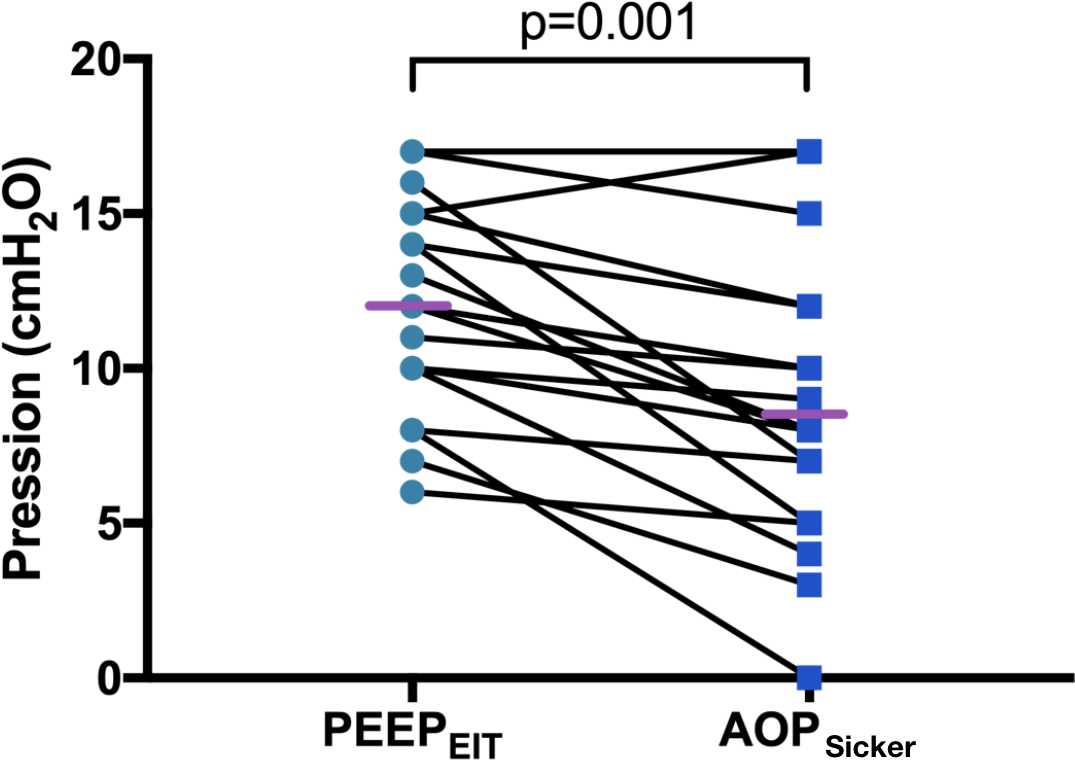

Supplement: Supplementary file 4 — Supplementary Material 4: This figure illustrates individual value of PEEPEIT during a decremental PEEP trial and the AOPsicker of the most injured lung [file 13613_2024_1379_MOESM4_ESM.tif]
